# Sub-Micromolar Methylmercury Exposure Promotes Premature Differentiation of Murine Embryonic Neural Precursor at the Expense of Their Proliferation

**DOI:** 10.3390/toxics6040061

**Published:** 2018-10-10

**Authors:** Xiaoyang Yuan, Jing Wang, Hing Man Chan

**Affiliations:** 1 Department of Biology, University of Ottawa, Ottawa, ON K1N 6N5, Canada; 2 Regenerative Medicine Program, Ottawa Hospital Research Institute, Ottawa, ON K1H 8L6, Canada; 3 Department of Cellular and Molecular Medicine, University of Ottawa, Ottawa, ON K1H 8M5, Canada; 4 Brain and Mind Research Institute, University of Ottawa, Ottawa, ON K1H 8M5, Canada

**Keywords:** methylmercury, cortical precursors, developmental neurotoxicity, low-dose exposure, delayed effects

## Abstract

Methylmercury (MeHg) is a ubiquitous environmental pollutant that is known to be neurotoxic, particularly during fetal development. However, the mechanisms responsible for MeHg-induced changes in adult neuronal function, when their exposure occurred primarily during fetal development, are not yet understood. We hypothesized that fetal MeHg exposure could affect neural precursor development leading to long-term neurotoxic effects. Primary cortical precursor cultures obtained from embryonic day 12 were exposed to 0 µM, 0.25 µM, 0.5 µM, 2.5 µM, and 5 µM MeHg for 48 or 72 h. All of the concentrations tested in the study did not affect cell viability. Intriguingly, we observed that cortical precursor exposed to 0.25 µM MeHg showed increased neuronal differentiation, while its proliferation was inhibited. Reduced neuronal differentiation, however, was observed in the higher dose groups. Our results suggest that micromolar MeHg exposure may deplete the pool of neural precursors by increasing premature neuronal differentiation, which can lead to long-term neurological effects in adulthood as opposed to the higher MeHg doses that cause more immediate toxicity during infant development.

## 1. Introduction

Methylmercury (MeHg) is a global pollutant affecting millions of people worldwide [1,2]. Its main target is the central nervous system, with fetuses being particularly susceptible [3]. Amin-Zaki et al. [4] reported that the levels of MeHg in fetal blood are about 25% higher than those of the mother. It has also been shown that fetuses can be affected in the absence of maternal toxicity [5].

A cohort study on the population of the Faroe Islands showed that prenatal exposure to MeHg was significantly associated with deficits in fine motor control, language, and learning abilities in children and adolescents [6]. Yorifuji et al. [7] demonstrated an increased prevalence of psychiatric symptoms in adults who showed no sign of toxicity at birth, based on an epidemiological study on the residents of Minamata.

Developmental exposure to MeHg in mice results in memory disturbances and induces depression-like behavior in adult animals [8], which persists into older age [9]. Animal studies have shown that the developing brain is extremely vulnerable to MeHg neurotoxicity [8,9,10], which may be attributed to the rapid cell proliferation [11] and cell differentiation in the developing brain [12,13]. Previous studies have demonstrated detrimental effects of MeHg at micromolar levels on neurogenesis, including inhibition of proliferation and disturbed cell cycle progression in neuronal cells [14].

The emerging idea of the “Developmental Origin of Health and Diseases (DOHaD)”, previously termed the "fetal origins of adult disease" in the 1990s, postulates that exposure to environmental influences during the embryonic period is related to the risk of developing diseases in adulthood [15]. Therefore, it is important to understand not only the immediate effect of MeHg exposure on the embryos themselves, but also its potential influences that progress into adulthood. The DOHaD theory suggests that epigenetic alterations could be induced by environmental conditions during development, which are maintained in adulthood. These subtle epigenetic changes, showing no effect in early ages, can increase the risk of developing diseases later in life [16]. Therefore, there is the necessity of understanding the effect of dietary MeHg exposure on very low levels of embryonic neural development and its underlying mechanisms.

In this study, we investigated the effects of low-dose (µM) MeHg exposure on regulating murine embryonic neural precursor development using a mouse cerebral cortex development model. Our hypothesis was that a low and non-toxic dose of MeHg could disrupt the development of these cortical precursors leading to long-term neurotoxic effects.

## 2. Materials and Methods

### 2.1. Animal Ethics

Animal care, handling and use protocols were reviewed and approved by the Animal Care Committee of the Ottawa Hospital Research Institute, University of Ottawa on 28 July, 2017. Animal protocol number: OHRI-2103.

### 2.2. Cell Culture Procedures and Experimental Treatments

Primary cultures of cortical precursors were obtained from embryonic day 12 cortices dissected in ice-cold Hanks’ balanced salt solution (HBSS) (Life Technologies, Carlsbad, CA, USA) from CD1 mouse (Charles River Laboratories, Wilmington, MA, USA). Embryos were transferred to ice-cold HBSS and the cerebral cortices were carefully isolated from the brain after removing the meninges. The tissue was mechanically triturated with a plastic pipette and plated at a density of 10^5^ cells on coverslips pre-coated with 15% poly-L-ornithine (PLO) (Sigma, St. Louis, MO, USA) and 5% laminin (BD Biosciences, Franklin Lakes, NJ, USA) in a 24-well plate (Thermo Scientific BioLite, Waltham, MA, USA). The cortical precursors were cultured in a neurobasal medium (Invitrogen, Carlsbad, CA, USA) containing 500 µM L-glutamine (Cambrex Biosciences, East Rutherford, NJ, USA), 2% B27 supplement (Invitrogen, Carlsbad, CA, USA), 1% penicillin-streptomycin (Invitrogen, Carlsbad, CA, USA), and 40 ng/ml FGF2 (BD Biosciences, Franklin Lakes, NJ, USA). The primary culture was exposed to 0 µM, 0.25 µM, 0.5 µM, 2.5 µM, and 5 µM MeHg for 48 or 72 h. The dosing solution was freshly prepared daily from a stock solution of 1 M using MeHgCl from Alfa Aesar (Ward Hill, MA, USA).

### 2.3. Immunocytochemistry

Cultured cells were fixed in 4% paraformaldehyde for 10 min, then blocked with 10% normal goat serum (NGS) diluted in PBS with 0.3% Triton X-100. Primary antibodies were diluted in 10% NGS in PBS with 0.3% Triton X-100 and incubated in a humid chamber at 4 °C overnight. Secondary antibodies were diluted in PBST and incubated for 1 h at room temperature. Hoechst 33258 was diluted in PBS. The culture was washed three times for 5 min with PBS between each step. The primary antibodies used were mouse anti-βIII-tubulin (1:1000; Covance, Princeton, NJ, USA), rabbit anti-Pax-6 (1:2000; BioLegend, San Diego, CA, USA), rabbit anti-Sox2 (1:100; Millipore, Burlington, MA, USA), mouse anti-Ki67 (1:400; Abcam, Cambridge, UK), and rabbit anti-Cleaved Caspase-3 (CC3) (1:400; Cell Signalling Tech., Danvers, MA, USA). The secondary antibodies used were Alexa Fluor 555- and Alexa Fluor 488-conjugated goat antibodies (1:500; Life Technologies, Carlsbad, CA, USA). Nuclear staining was performed with Hoechst 33343 (1:1000; Sigma, St. Louis, MO, USA). After rinsing with PBS, the coverslips were mounted in a Lab Vision PermaFluor Aqueous Mounting Medium (Thermo Fisher, Waltham, MA, USA). All experiments were repeated at least three times.

### 2.4. Microscopy and Quantification

Digital image acquisition was performed using a Zeiss Axioplan 2 fluorescent microscope with Zeiss Axiovision software (Carl Zeiss Microscopy, Thornwood, NY, USA). Six random images over 300 cells per condition per experiment were taken for quantification. 

### 2.5. Statistics

All data were expressed as the mean plus or minus the standard error of the mean (SEM) and were tested for statistical significance with one-way ANOVA, followed by Bonferroni’s post hoc test. All statistical analyses were performed using Prism (version 7, GraphPad Software, La Jolla, CA, USA, 2018). 

## 3. Results

### 3.1. Cell Viability

**Figure 1:**
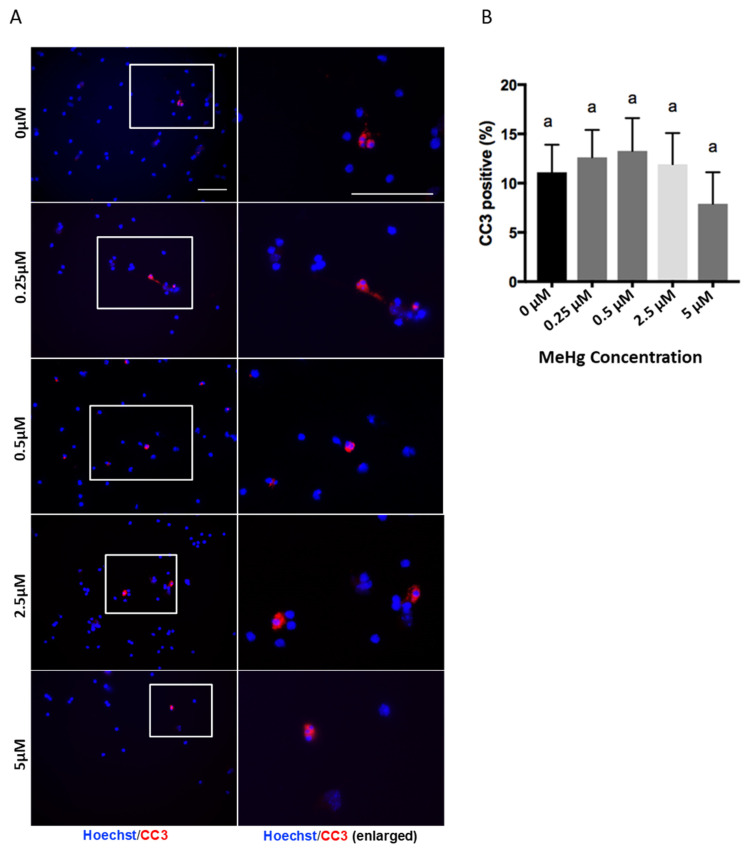
Methylmercury treatments at doses of 0.25 to 5 µM do not induce cell death. Immunocytochemistry was performed in a 2-day cortical precursors culture. Images of Cleaved Caspase-3 (CC3) (Red) and Hoechst (Blue) staining are shown. Scale bar = 50 μm (**A**). The bar graph (**B**) shows the percentage of immunocytochemistry-positive cells. Values are mean ± SEM (*n* = 3). Statistical significance was determined by a one-way ANOVA followed by a Bonferroni’s post hoc test. No significant *p*-value was obtained for ANOVA. Letters denote the results of the comparisons between treatment groups, and groups with the same letter were not statistically different.

Immunocytochemistry results showed that the percentage of CC3+ condensed nuclei was not changed between the methylmercury treated groups and the control group (Figure 1A). One-way ANOVA results showed that MeHg treatments did not have any effect on CC3+, F = 0.4666, d.f. = (4,10), *p* > 0.05) (Figure 1B). Thus, the exposure levels of methylmercury used in our experiments did not the affect cell viability of the cortical precursors.

### 3.2. Effects of Cortical Precursors on Proliferation and Differentiation

**Figure 2:**
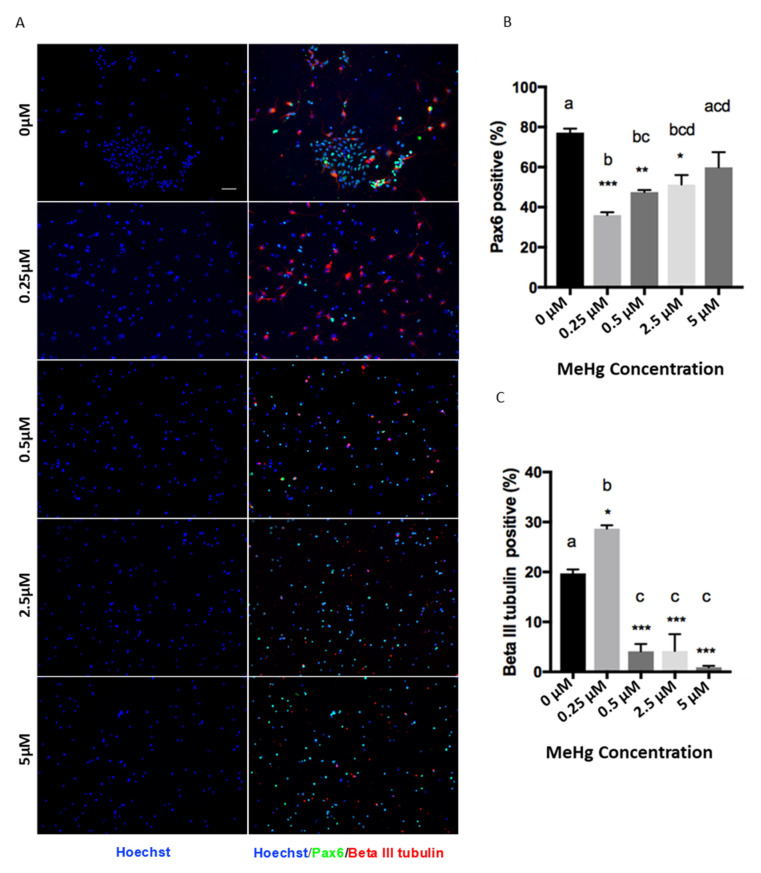

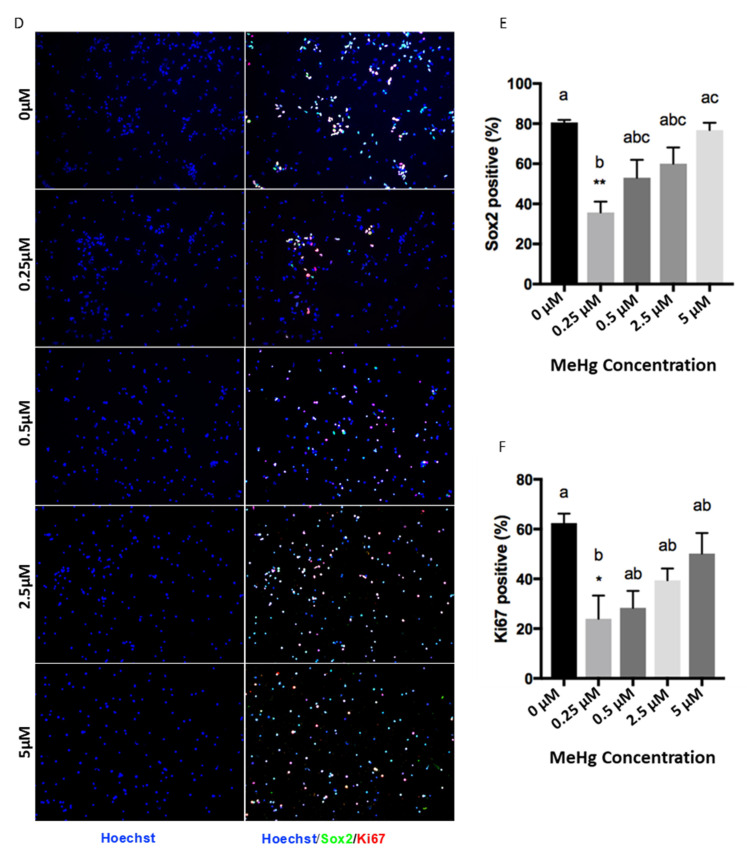
Immunocytochemistry was performed in a 3-day NSC culture. Images of (**A**) Pax6 (Green), Beta III Tubulin (Red), (**D**) Sox2 (Green), Ki67 (Red), and Hoechst (Blue) staining are shown. Scale bar = 50 μm. The bar graphs (**B**,**C**; **E**,**F**) show the percentage of immunocytochemistry-positive cells. Values are mean ± SEM (*n* = 3). Statistical significance was determined by a one-way ANOVA followed by a Bonferroni’s post hoc test (* *p* < 0.05, ** *p* < 0.01, *** *p* < 0.001: vs. control, letters denote the results of the comparisons between treatment groups, groups with the same letter were not statistically different).

The MeHg treatments had a significant effect on the proliferation and differentiation of cortical precursors. One-way ANOVA results showed that there was a significant MeHg treatment effect for Pax6 (F = 13.56, d.f. = (4,10), *p* < 0.05) and β III tubulin staining (F = 50.12, d.f. = (4,10), *p* < 0.05). Immunofluorescence results showed that exposure to 0.25 µM MeHg significantly increased the percentage of newborn neurons produced from E12 cortical precursors, labeled with βIII tubulin, compared to the control (Figure 2A). Coincidentally, the population of Pax6 + cortical precursors was significantly decreased at 0.25 µM MeHg (Figure 2B). To validate the reduced pool of cortical precursors in the culture, we performed immunocytochemistry analysis with a pan-neural stem cell marker, Sox2, and a cell cycling marker, Ki67. One-way ANOVA results showed that there was a significant MeHg treatment effect for Sox2 (F = 8.849, d.f. = (4,10), *p* < 0.05) and Ki67 staining (F = 5.182, d.f. = (4,10), *p* < 0.05). The results showed that both the percentage of Sox2+ cortical precursors and Ki67+ cycling cells were dramatically decreased upon exposure to 0.25 µM MeHg (Figure 2D–F). These results suggest that exposure of cortical precursors to an extremely low dose (0.25 µM) of MeHg enhanced premature neuronal differentiation while reducing their proliferation. In comparison, exposure of cortical precursors to MeHg from 0.5 µM to 5 µM reduced the percentage of βIII tubulin+ newborn neurons in culture (Figure 2A,C). However, while the 0.5 µM and 2.5 µM treatment groups showed a significant decrease in the population of Pax6+ cortical precursors, the 5 µM treatment group did not (Figure 2B). In addition, the percentage of Sox2+ NSCs and Ki67+ cycling cells were not changed in the 0.5 µM and 5 µM MeHg treatment groups (Figure 2D–F). These results show that exposure to 0.5 µM did not show the effects that we observed at the lower dose of 0.25 µM. Its effect was more similar to the effects observed at 2.5 and 5 µM MeHg, which showed significantly lower neuronal differentiation, while its proliferation recovered gradually to a level comparable with the control.

## 4. Discussion

The novel finding of this study is that the effects of MeHg on cortical precursor development are dose-dependent. The extremely low micromolar dose of 0.25 µM MeHg increases the neuronal differentiation of cortical precursors while reducing their proliferation. On the other hand, there was a decrease in differentiation at higher doses (>0.5 µM). These reduced differentiation phenotypes were reported in the existing literature with lower dose application. MeHg (2.5–5 nM MeHg for 48 h) were shown to inhibit the spontaneous neuronal differentiation of murine embryonic neural stem cells [12]. Fujimura & Usuki [17] also showed that neural progenitor cell proliferation was suppressed 48 h after exposure to 10 nM MeHg, but cell death was not observed. Tamm et al. [18] identified Notch signaling as a target for methylmercury’s inhibition of neuronal differentiation at exposure levels between 2.5 and 10 nM. Bose et al. [19] exposed E15 primary cultures of rat embryonic cortical neural stem cells to 2.5 nM and 5 nM MeHg for 48 h and reported reduced cell proliferation with no effect on the cell death rate. The discrepancy between our study and early work in terms of MeHg dose-response might be due to variations among the selected culture model and the rodent species used.

Our new findings on the increase in premature differentiation during embryonic development at a sub-nanomolar MeHg dose might be an outcome of epigenetic changes triggered by stress sensors, such as AMP dependent kinase (AMPK). Even though it is not known whether MeHg can increase AMPK activity, HgCl_2_ has been shown to enhance AMPK activation in the liver of mice [20]. Hwang et al. [21] have shown that the activation (phosphorylation) of AMPK can play an important role in reducing the toxicity of methylmercury. Therefore, the activation of AMPK could be a biological response induced by the extremely low dose of MeHg. Our previous work has shown that AMPK activation can stimulate a signaling-directed epigenetic pathway, atypical protein kinase C (aPKC)-mediated S436 phosphorylation of CREB-binding protein and histone acetyltransferase, to promote the neuronal differentiation of embryonic and adult neural precursor cells [22,23]. Moreover, it has been reported that the levels of cysteine and glutathione (GSH) as well as the GSH/GSSG ratio in neural stem cells progressively decreased in association with neuronal differentiation [24]. Since it is well known that MeHg decreases GSH, this may also be a potential mechanism for the observed effects.

This enhanced neuronal differentiation by sub-nanomolar MeHg could have significant biological consequences. The untimely enhancement of embryonic neurogenesis can lead to depletion of the neural precursor cell pool and consequently a decreased level of adult neurogenesis resulting in neurological functional impairment. Juliandi et al. [25] have shown that prenatal treatment of valproic acid in mice can enhance neurogenesis and reduce the proliferation of neural precursor cells (NPCs), leading to the depletion of the NPC pool. This depletion may cause a slower differentiation of the residual NPCs during life. In contrast, Gallaher et al. [26] showed that a maternal IL-6 surge aberrantly affected embryonic precursors, ultimately causing an expanded adult forebrain NPC pool and enhanced olfactory neurogenesis in offspring, months after fetal exposure. The possibility that different doses of prenatal exposure to MeHg can have different effects on the adult NPC pool and its associated neurological effects is an interesting avenue to be explored in the future.

While the doses used in this in-vitro experiment cannot be extrapolated to MeHg doses during human fetal development, the difference of effects observed between the sub-nanomolar range and the nanomolar range has biological significance. An analysis of autopsied brain tissue from infants prenatally exposed to methylmercury showed that the mercury levels detected were 0.026–0.295 μg/g [27]. Sakamoto et al. [28] measured total Hg concentrations in the cord blood of 54 healthy Japanese pregnant women, with no particular exposure to any Hg compounds at Fukuda Hospital (Kumamoto City, Kumamoto, Japan) from 2006 to 2007, and reported a mean Hg concentration of 7.26 ng/g. This means that the nM range of exposure is environmentally relevant.

**Figure 3:**
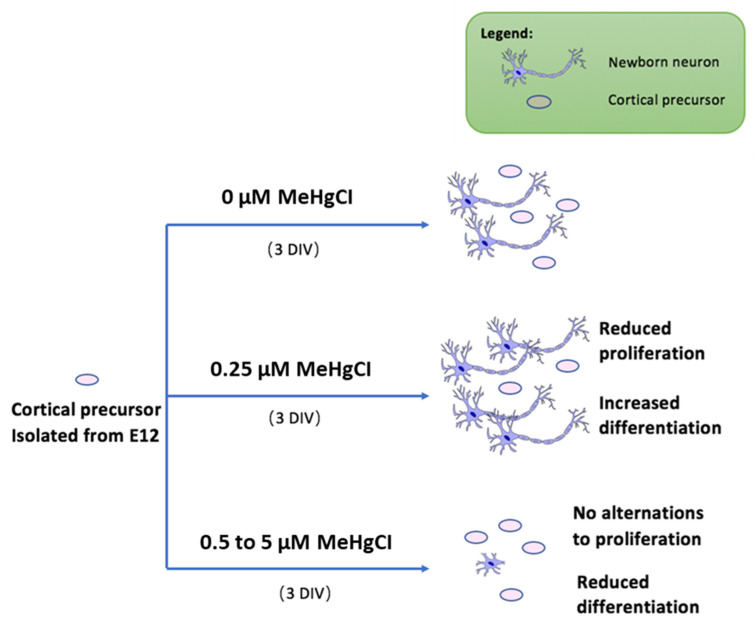
Proposed model for low-dose MeHg exposure to NSCs. Under normal circumstances, cortical precursor cells undergo both differentiation and proliferation. Upon administration of 0.25 µM MeHgCl, the cell population showed reduced proliferation and increased differentiation. Cell population dosed with 0.5 µM to 5 µM MeHgCl shows inhibited differentiation and gradual recovery of proliferation.

In conclusion, based on our results, we propose a novel model for low-dose MeHg exposure to neural precursor cells, as shown in Figure 3. Extremely low-dose MeHg exposure may also have a detrimental effect on embryonic neural development, which may lead to neurodevelopmental disorders or neurodegeneration later in life.
