# Correction: Yuan et al. Sub-Micromolar Methylmercury Exposure Promotes Premature Differentiation of Murine Embryonic Neural Precursor at the Expense of Their Proliferation. *Toxics* 2018, *6*, 61

**DOI:** 10.3390/toxics9120322

**Published:** 2021-11-26

**Authors:** Xiaoyang Yuan, Jing Wang, Hing Man Chan

**Affiliations:** 1 Department of Biology, University of Ottawa, Ottawa, ON K1N 6N5, Canada; michelle_yuan@hotmail.com; 2 Regenerative Medicine Program, Ottawa Hospital Research Institute, Ottawa, ON K1H 8L6, Canada; JIWang@ohri.ca; 3 Department of Cellular and Molecular Medicine, University of Ottawa, Ottawa, ON K1H 8M5, Canada; 4 Brain and Mind Research Institute, University of Ottawa, Ottawa, ON K1H 8M5, Canada

## 1. Incorrect Title

In the original article [1], there is an error in the title. The correct title of the article is “Sub-Micromolar Methylmercury Exposure Promotes Premature Differentiation of Murine Embryonic Neural Precursor at the Expense of Their Proliferation”. 

## 2. Incorrect Text

In the original article [1], the reported MeHg dosing concentrations were decreased by 1000 times. The correct concentrations in the text should be increased by 1000 times. 

Starting with the fourth sentence of the Abstract, the correct sentences are: Primary cortical precursor cultures obtained from embryonic day 12 were exposed to 0 µM, 0.25 µM, 0.5 µM, 2.5 µM, and 5 µM MeHg for 48 or 72 h. All of the concentrations tested in the study did not affect cell viability. Intriguingly, we observed that cortical precursor exposed to 0.25 µM MeHg showed increased neuronal differentiation, while its proliferation was inhibited. Reduced neuronal differentiation, however, was observed in the higher dose groups. Our results suggest that micromolar MeHg exposure may deplete the pool of neural precursors by increasing premature neuronal differentiation, which can lead to long-term neurological effects in adulthood as opposed to the higher MeHg doses that cause more immediate toxicity during infant development.

In last paragraph of Introduction Section, “nM” should be “µM”

In line 12 of Section 2.2, “0 nM, 0.25 nM, 0.5 nM, 2.5 nM, and 5 nM MeHg” should be “0 µM, 0.25 µM, 0.5 µM, 2.5 µM, and 5 µM MeHg”.

Sentences “A two-tailed Student’s *t*-test was used for between-group comparisons. The differences were considered significant if *p* < 0.05.” should be deleted from Section 2.5. 

In Section 3.2, the whole paragraph should be replaced with “The MeHg treatments had a significant effect on the proliferation and differentiation of cortical precursors. One-way ANOVA results showed that there was a significant MeHg treatment effect for Pax6 (F = 13.56, d.f. = (4,10), *p* < 0.05) and β III tubulin staining (F = 50.12, d.f. = (4,10), *p* < 0.05). Immunofluorescence results showed that exposure to 0.25 µM MeHg significantly increased the percentage of newborn neurons produced from E12 cortical precursors, labeled with βIII tubulin, compared to the control (Figure 2A). Coincidentally, the population of Pax6 + cortical precursors was significantly decreased at 0.25 µM MeHg (Figure 2B). To validate the reduced pool of cortical precursors in the culture, we performed immunocytochemistry analysis with a pan-neural stem cell marker, Sox2, and a cell cycling marker, Ki67. One-way ANOVA results showed that there was a significant MeHg treatment effect for Sox2 (F = 8.849, d.f. = (4,10), *p* < 0.05) and Ki67 staining (F = 5.182, d.f. = (4,10), *p* < 0.05). The results showed that both the percentage of Sox2+ cortical precursors and Ki67+ cycling cells were dramatically decreased upon exposure to 0.25 µM MeHg (Figure 2D–F). These results suggest that exposure of cortical precursors to an extremely low dose (0.25 µM) of MeHg enhanced premature neuronal differentiation while reducing their proliferation. In comparison, exposure of cortical precursors to MeHg from 0.5 µM to 5 µM reduced the percentage of βIII tubulin+ newborn neurons in culture (Figure 2A,C). However, while the 0.5 µM and 2.5 µM treatment groups showed a significant decrease in the population of Pax6+ cortical precursors, the 5 µM treatment group did not (Figure 2B). In addition, the percentage of Sox2+ NSCs and Ki67+ cycling cells were not changed in the 0.5 µM and 5 µM MeHg treatment groups (Figure 2D–F). These results show that exposure to 0.5 µM did not show the effects that we observed at the lower dose of 0.25 µM. Its effect was more similar to the effects observed at 2.5 and 5 µM MeHg, which showed significantly lower neuronal differentiation, while its proliferation recovered gradually to a level comparable with the control”. 

In Section 4, the correct text of the first paragraph is: The novel finding of this study is that the effects of MeHg on cortical precursor development are dose-dependent. The extremely low micromolar dose of 0.25 µM MeHg increases the neuronal differentiation of cortical precursors while reducing their proliferation. On the other hand, there was a decrease in differentiation at higher doses (>0.5 µM). These reduced differentiation phenotypes were reported in the existing literature with lower dose application. MeHg (2.5–5 nM MeHg for 48 h) were shown to inhibit the spontaneous neuronal differentiation of murine embryonic neural stem cells [12]. Fujimura & Usuki [17] also showed that neural progenitor cell proliferation was suppressed 48 h after exposure to 10 nM MeHg, but cell death was not observed. Tamm et al. [18] identified Notch signaling as a target for methylmercury’s inhibition of neuronal differentiation at exposure levels between 2.5 and 10 nM. Bose et al. [19] exposed E15 primary cultures of rat embryonic cortical neural stem cells to 2.5 nM and 5 nM MeHg for 48 h and reported reduced cell proliferation with no effect on the cell death rate. The discrepancy between our study and early work in terms of MeHg dose-response might be due to variations among the selected culture model and the rodent species used. 

## 3. Incorrect Figures and Legends

In the original article [1], there were mistakes in the labels and legends of Figure 1, Figure 2 and Figure 3. All concentrations should be multiplied by 1000. The correct figures and legends appear below. 

The authors would like to apologize for any inconvenience caused to the readers by these changes. The original article has been updated. The changes do not affect the scientific results.

## Figures and Tables

**Figure 1:**
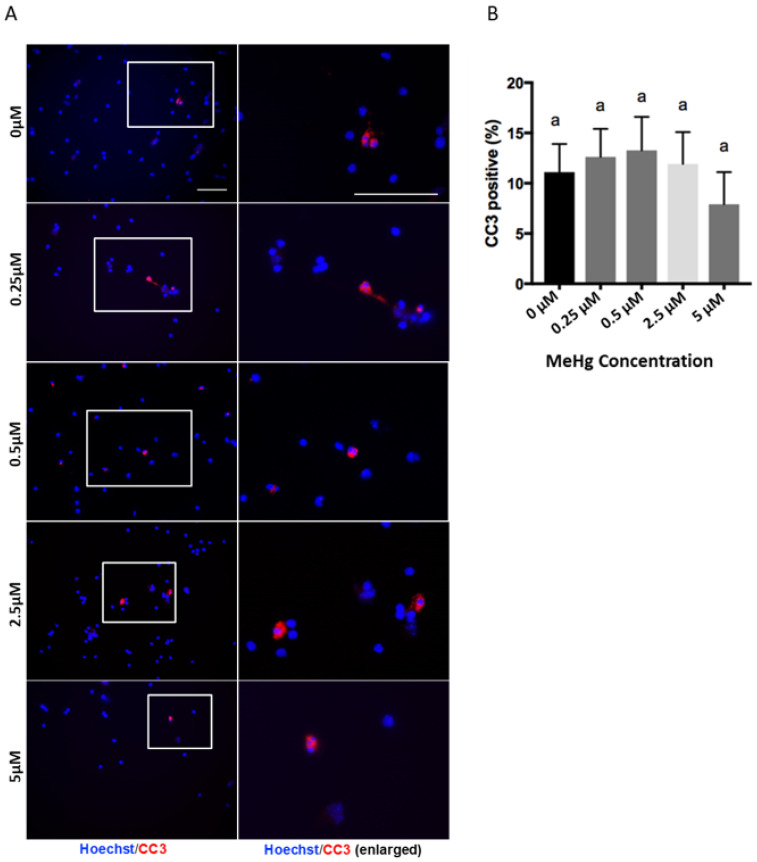
Methylmercury treatments at doses of 0.25 to 5 µM do not induce cell death. Immunocytochemistry was performed in a 2-day cortical precursors culture. Images of Cleaved Caspase-3 (CC3) (Red) and Hoechst (Blue) staining are shown. Scale bar = 50 µm (**A**). The bar graph (**B**) shows the percentage of immunocytochemistry-positive cells. Values are mean ± SEM (*n* = 3). Statistical significance was determined by a one-way ANOVA followed by a Bonferroni’s post hoc test. No significant *p*-value was obtained for ANOVA. Letters denote the results of the comparisons between treatment groups, and groups with the same letter were not statistically different.

**Figure 2:**
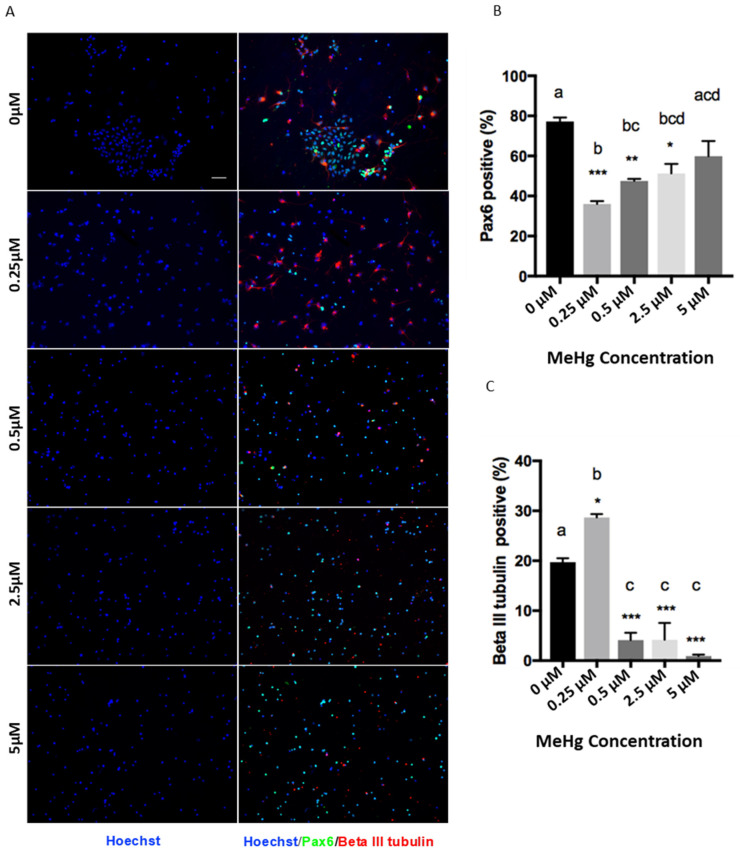

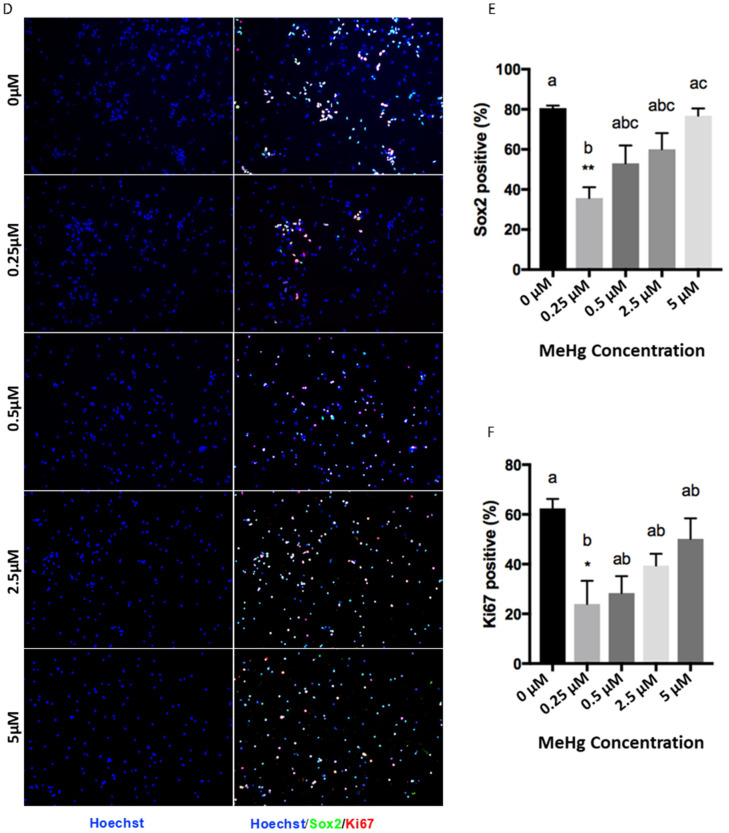
Immunocytochemistry was performed in a 3-day NSC culture. Images of (**A**) Pax6 (Green), Beta III Tubulin (Red), (**D**) Sox2 (Green), Ki67 (Red), and Hoechst (Blue) staining are shown. Scale bar = 50 µm. The bar graphs (**B**,**C**,**E**,**F**) show the percentage of immunocytochemistry-positive cells. Values are mean ± SEM (*n* = 3). Statistical significance was determined by a one-way ANOVA followed by a Bonferroni’s post hoc test (* *p* < 0.05, ** *p* < 0.01, *** *p* < 0.001: vs. control, letters denote the results of the comparisons between treatment groups, groups with the same letter were not statistically different).

**Figure 3:**
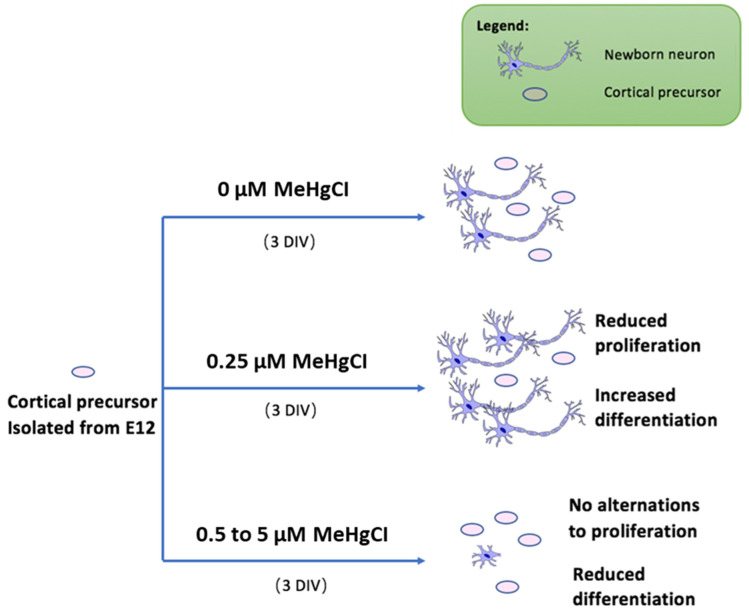
Proposed model for low-dose MeHg exposure to NSCs. Under normal circumstances, cortical precursor cells undergo both differentiation and proliferation. Upon administration of 0.25 µM MeHgCl, the cell population showed reduced proliferation and increased differentiation. Cell population dosed with 0.5 µM to 5 µM MeHgCl shows inhibited differentiation and gradual recovery of proliferation.
